# Community-acquired lobar pneumonia in children in the era of universal 7-valent pneumococcal vaccination: a review of clinical presentations and antimicrobial treatment from a Canadian pediatric hospital

**DOI:** 10.1186/1471-2431-12-133

**Published:** 2012-08-28

**Authors:** Anne Rowan-Legg, Nicholas Barrowman, Nazih Shenouda, Khaldoun Koujok, Nicole Le Saux

**Affiliations:** 1 Department of Pediatrics, Children’s Hospital of Eastern Ontario, Ottawa, Canada; 2 Clinical Research Unit, Children’s Hospital of Eastern Ontario Research Institute, Ottawa, Canada; 3 Department of Diagnostic Imaging, Children’s Hospital of Eastern Ontario, Ottawa, Canada

## Abstract

**Background:**

Community-acquired pneumonia (CAP) is a common cause of pediatric admission to hospital. The objectives of this study were twofold: 1) to describe the clinical characteristics of CAP in children admitted to a tertiary care pediatric hospital in the pneumococcal vaccination era and, 2) to examine the antimicrobial selection in hospital and on discharge.

**Methods:**

A retrospective review of healthy immunocompetent children admitted to a tertiary pediatric hospital from January 2007 to December 2008 with clinical features consistent with pneumonia and a radiographically-confirmed consolidation was performed. Clinical, microbiological and antimicrobial data were collected.

**Results:**

One hundred and thirty-five hospitalized children with pneumonia were evaluated. Mean age at admission was 4.8 years (range 0–17 years). Two thirds of patients had been seen by a physician in the 24 hours prior to presentation; 56 (41.5%) were on antimicrobials at admission. 52 (38.5%) of patients developed an effusion, and 22/52 (42.3%) had pleural fluid sampled. Of 117 children who had specimens (blood/pleural fluid) cultured, 9 (7.7%) had pathogens identified (7 Streptococcus pneumoniae, 1 Group A Streptococcus, and 1 Rhodococcus). 55% of patients received 2 or more antimicrobials in hospital. Cephalosporins were given to 130 patients (96.1%) in hospital. Only 21/126 patients (16.7%) were discharged on amoxicillin. The median length of stay was 3 days (IQR 2–4) for those without effusion and 9 (IQR 5–13) for those with effusion. No deaths were related to pneumonia.

**Conclusions:**

This study provides comprehensive data on the clinical characteristics of hospitalized children with CAP in the pneumococcal 7-valent vaccine era. Empiric antimicrobial choice at our institution is variable, highlighting a need for heightened antimicrobial stewardship.

## Background

Pediatric community-acquired pneumonia (CAP) is a common and potentially serious childhood infection, which often results in hospital admission. The World Health Organization has estimated that in developed countries, one in twenty children under the age of five years will have an episode of pneumonia each year, and 1 to 4 per 1000 children are admitted to hospital annually [1]*. S. pneumoniae* continues to be the most important pathogen in bacterial pneumonia. The 7-valent pneumococcal conjugate vaccine (Prevnar®, PCV-7) became available in Canada in 2000, and was added to the routine Ontario immunization schedule in January 2005 [2]. Introduction of the pneumococcal vaccine has been shown to decrease all-cause radiologic pneumonia admission rates by an average of 27% in children under the age of 5 years [3-10].

The diagnostic and therapeutic approach to pneumonia in children is not standardized. The diagnosis of CAP in hospitalized children often relies on a chest radiograph showing consolidation (alveolar infiltrates) or consolidation complicated by effusion. It has been suggested that a radiographic pattern of alveolar infiltrates is most frequently associated with bacterial infection [11]. Available clinical guidelines for empiric treatment of pediatric CAP are largely based on consensus opinion [12-14] This likely relates to low rates of pathogen identification, the spectrum of severity, and a paucity of randomized controlled trials to guide antimicrobial therapy. Without evidence-based guidelines, antimicrobial treatment of pediatric CAP has been shown to be variable in both inpatient and outpatient settings, with a tendency to overuse broad spectrum antimicrobials [15-18].

We conducted a retrospective study of healthy children admitted to hospital with CAP (based on presentation of consolidative pneumonia and compatible clinical symptoms) to describe their clinical course and management, in the era of PCV-7 vaccination.

## Methods

### Study location

The Children’s Hospital of Eastern Ontario (CHEO, Ottawa, Canada) is an urban, tertiary pediatric care centre with a catchment population of over 2,000,000 people (eastern Ontario, western Quebec and Baffin Island) [19]. It is the only hospital in the catchment area that admits children. The Children’s Hospital of Eastern Ontario does not have empiric management guidelines for CAP.

### Study design

A retrospective chart review of admitted children with ICD-10 discharge codes of ‘pneumonia’, ‘empyema’, ‘parapneumonic effusion’, ‘pyothorax’, or ‘pleural effusion’ from January 1, 2007 to December 31, 2008 was conducted. We excluded any child that had an associated underlying condition coded at the time of the pneumonia.

The admitting chest radiograph (CXR) reports (as reported by pediatric radiologists) were classified by 2 independent reviewers (AR-L and NLS) as reporting: i) definite consolidation, ii) no consolidation, or iii) ‘possible’ consolidation. Only cases that had definite consolidation, or consolidation associated with an effusion, were included for further review.

Two pediatric radiologists independently reviewed a sample of 31 of the CXRs (without access to the CXR report) and were asked to indicate whether the CXR had a definite consolidation. (Of these 31 cases, 6 had been previously excluded by AR-L/NS on the basis of an equivocal or negative CXR report for consolidation, and 25 were a random sample of those included in the final study analysis.) Kappa values were calculated to ensure agreement between reviewers.

Each chart was subsequently reviewed to determine if the patient met the study inclusion criteria: i) age 0–18 years, and ii) no underlying chronic illnesses that would predispose to aspiration or pneumonia. Exclusion criteria were as follows: i) presence of a comorbid condition [e.g. hemodynamically significant heart disease, malignancy, hemoglobinopathy, underlying pulmonary pathology (e.g. cystic fibrosis, bronchiectasis or bronchopulmonary dysplasia), cerebral palsy or other chronic neurological disease, upper airway mechanical problems (e.g. tracheostomy, stenosis), or genetic syndrome], or ii) a clinical history incompatible with pneumonia. Neither asthma nor prematurity were considered exclusion criteria.

A standardized case report form was developed to collect demographic, clinical, diagnostic, treatment and outcome data on all cases meeting inclusion criteria. The medical record was systematically reviewed by one of four independent reviewers (AR-L, NL, CG, MB). Hypoxia was defined as an oxygen saturation ≤ 92%. Tachypnea was defined according to the World Health Organization definition: <2 months: respiratory rate (RR) ≥60 breaths/minute, 2 – 12 months: RR ≥50 breaths/minute, 1–5 years: RR ≥40 breaths/minute and >5 years: RR >30 breaths/minute [20]. A large effusion was defined as involving ≥ 2/3 of the hemithorax.

The total length of antimicrobial therapy included the number of days of antimicrobials administered in hospital in addition to those prescribed at discharge. The principal antimicrobial used in hospital was defined as the antimicrobial(s) given for the longest time period in hospital.

Discrete variables were summarized using frequency and percentage. Continuous variables were summarized using mean and standard deviation or median and interquartile range, as appropriate. Proportions were compared using Pearson’s chi-square test or Fisher’s exact test, as appropriate. Association between patient age and presence of an effusion was assessed using binary logistic regression. Kappa values were calculated between the two radiologists, and between AR-L/NS and each of the two radiologists separately. All analyses were performed using SPSS version 19.0.2 [21].

The study was approved by the Research Ethics Board at the Children’s Hospital of Eastern Ontario (2009).

## Results

### Study population

Two-hundred and thirty-two patients had eligible ICD-10 discharge codes for entry into the study. Of those, 143 patients met the criteria of having a definite consolidation on CXR report. In a sample of 31/143 charts, moderate agreement (k = 0.52) was found between the judgments of two independent radiologists (NS and KK) for the presence of consolidation. The agreement between AR-L/NS and one radiologist was substantial (k = 0.67) and with the other radiologist, the agreement was moderate (k = 0.44) [22].

After chart review, 135 patients met inclusion criteria. The reasons for patient exclusion were: 5 had underlying conditions (e.g. cerebral palsy, alpha-1-antitrypsin deficiency, hepatitis A, and congenital cystic adenomatoid malformation), and 3 had a clinical history incompatible with pneumonia (i.e. respiratory symptoms on hospital admission were better accounted for by conditions such as severe reactive airway disease, croup, or aspiration). See Table 1 for demographic characteristics of the study population.

**Table 1:** Demographic characteristics of study population (n = 135)

	
Age at admission (years); mean (SD) [range]	4.8 (3.9) [0–17 years]
Sex (male); n (%)	76 (56.3)
Prematurity (gestational age <37 weeks); n (%)	9 (6.7)
Month of admission: n (%)	
January – March	47 (34.8)
April – June	30 (22.2)
July – September	25 (18.5)
October – December	33 (24.4)
Length of history of symptoms (days); median (IQR)	
Patients without effusion	4 (3–7)
Patients with effusion	7 (5–10)
Fever prior to presentation; n (%)	127 (94.1)
Previous visit to physician in prior 24 hours documented; n (%)	86 (63.7)
Antimicrobials prescribed prior to presentation documented; n (%)	56 (41.5)
Hospitalization for asthma in past year; n (%)	3 (2.2)
Attendance at daycare or school documented; n (%)	100 (74.1)
Up to date immunizations documented; n (%)	120 (88.9)
Admitted from: n (%)	
CHEO Emergency Department	108 (80)
Transferred from peripheral hospital as outpatient	17 (12.6)
Transferred from peripheral hospital as inpatient	10 (7.4)

The most common pre-admission clinical signs were fever (94.1%), cough (89.6%), decreased oral intake (71.9%), vomiting (53.3%) and nasal congestion (49.6%). The clinical features of the course in hospital are outlined in Table 2.

**Table 2:** Clinical course in hospital of patients admitted with pneumonia (n = 135)

	
Fever (≥38.0°C) at triage; n (%)	51 (37.8)
Hypoxia (O_2_ saturation ≤92%) at triage; n (%)	31 (23.0)
Tachypnea (WHO classification) at triage; n (%)	61 (45.2)
Initial white blood cell count (x 109/L); mean (SD) [range] – median (IQR)	17.23 (9.64) [1.29-54.2] – 14.9 (9.6-23.1)
Days of fever (≥38.0°C); in hospital; mean (SD)	1.88 (2.58)
Developed a supplemental O_2_ need in hospital; n (%)	44 (32.6)
Days requiring supplemental O_2_ in those children requiring O_2_ therapy; mean (SD)	3.92 (4.13)
Length of stay (days): median (IQR)	
All patients	4 (3–8)
Patients without effusion	3 (2–4)
Patients with effusion	9 (5–13)
Admission to intensive care unit; n (%)	13 (9.6)
Deaths; n (%)	0 (0)

Of the 135 patients, 75 (56%) had one CXR, 24 (18%) had two CXRs, and 34 (25%) had ≥ 3 CXRs. Two patients had an initial CXR done at an external hospital. One patient (admitted to the intensive care unit) had 29 CXRs done.

### Pneumonia with effusion

Effusions were identified on CXR in 52 (38.5%) patients; 46 (34.1%) were identified on admission and 6 developed in hospital.

Patients with effusions had a longer duration of pre-admission symptomatology (7 vs 4 days) but were not more likely to be febrile (p = 0.58), tachypneic (p = 0.58), or have a higher white blood cell count (p = 0.90) compared to those without effusion. The presence of an effusion was not associated with age (p = 0.89). Patients with effusions had a longer length of stay compared to children with uncomplicated pneumonia (9 vs 3 days).

Chest ultrasound was performed in 28/52 (53.8%) of patients with effusion, and 16/52 (30.8%) had computerized tomography of the chest for diagnostic purposes. Of this group, 23/52 (44.2%) had a chest tube or pigtail catheter inserted and 14/52 (26.9%) underwent a video-assisted thoracoscopic (VATS) procedure.

Pleural fluid was sampled in 22/52 (42.3%) of patients with an effusion a median of 4 days after admission (IQR 1–6). All samples were obtained after antimicrobials had been started.

Of the patients without effusion, 3/83 (3.6%) were admitted to the ICU, whereas 10/52 (19.2%) of patients with effusion were admitted to the ICU, showing that the presence of an effusion is significantly associated with ICU admission (p = 0.005).

### Microbiology

#### Bacteremic pneumonia

7 (6.0%) of 117 patients who had blood cultures taken had a bacteremic pneumonia; 6 (86%) grew *S. pneumoniae* and 1 grew *Rhodococcus* species (5 year-old immunocompetent girl with acute onset of fever and consolidative pneumonia). Additionally, 2 blood cultures grew presumed contaminants (alpha-hemolytic *Streptococcus*, *Staphylococcus epidermidis*).

#### Pleural fluid

Of 22 patients who had pleural fluid sampled, 3 (13.6%) had a positive gram stain (1 Gram positive cocci and Gram negative cocci, and 2 Gram positive cocci) while 2 (9.1%) had a positive pleural fluid culture (Group A *Streptococcus* and *S. pneumoniae*). These latter 2 patients were not bacteremic.

Of the 7 identified *S. pneumoniae* serotypes in blood or pleural fluid, three were 19A (one from pleural fluid, two from blood), two were serotype 3, one was 11A, and one was 9 V.

#### *Mycoplasma* testing

Of 12 (8.9%) patients tested for *Mycoplasma* by throat swab PCR 3/12 (25%) were positive. 46 (34.1%) were tested for *Mycoplasma* by IgM serology and 13 (28.3%) were positive.

#### Viral testing

Throat or nasopharyngeal swab for viral culture or antigen detection was done in 61 patients (45.1%), and 7 (11.5%) were positive: 5 with respiratory syncytial virus, 1 enterovirus, and 1 herpes simplex virus Type 1.

### Antimicrobial usage

The most frequently prescribed combinations of antimicrobials during hospitalization are summarized in Table 3.

**Table 3:** Frequency of principal antimicrobial use during hospital admission (n = 135)

**Antimicrobial (s)**	**Frequency (n = 135)**
Cefuroxime; n (%)	60 (44.4)
Cefuroxime and Clarithromycin; n (%)	36 (26.7)
Cefuroxime and Clindamycin; n (%)	15 (11.1)
Ampicillin; n (%)	2 (1.5)
Penicillin; n (%)	3 (2.2)
Ceftriaxone and Clindamycin; n (%)	4 (2.9)
Clindamycin and Clarithromycin; n (%)	2 (1.5)
Ceftriaxone; n (%)	1 (0.7)
Clarithromycin; n (%)	1 (0.7)
Other; n (%)	11 (8.1)

Of the patients that had documented *S. pneumoniae* (all penicillin susceptible) in a blood or pleural fluid sample, 2 received penicillins, 2 received vancomycin and a third generation cephalosporin, 1 received ceftriaxone, 1 received cefuroxime and clindamycin, and 1 received cefuroxime alone. At discharge, 2 of these patients received amoxicillin, 3 patients received amoxicillin-clavulinic acid, 1 was discharged without antimicrobials, and 1 continued intravenous ceftriaxone (to treat a lung abscess).

Of the 83 patients with uncomplicated pneumonia, 40 (48.2%) received 1 antimicrobial, 33 (39.8%) received 2, 8 (9.6%) received 3, and 2 (2.4%) received four antimicrobials (simultaneously or consecutively) while in hospital. Of the 52 patients with an effusion, 7 (13.5%) received 1 antimicrobial, 20 (38.5%) received 2, 14 (26.9%) received 3, 7 (13.5%) received four, and 4 (7.7%) received 5 antimicrobials over the course of their stay.

Only 47 patients (34.8%) received one antimicrobial during admission; 46 (97.9%) received cefuroxime and one received clarithromycin. A third generation cephalosporin was given to 15 (11.1%) of children. A macrolide was used in hospital or at discharge in 77/135 (57.0%) of patients. Patients under 5 years of age received a macrolide in hospital or at discharge in 38/83 (45.8%) of cases, compared to 39/52 (75.0%) of patients 5 years and older (p = 0.001).

The antimicrobials prescribed at discharge for patients with and without effusion are included in Table 4.

**Table 4:** Discharge antimicrobials for patients without effusion (n = 83) and with effusion (n = 52)

	**Without effusion (n = 83)**	**With effusion (n = 52)**
No Antimicrobials; n (%)	5 (6.0)	4 (7.7)
Amoxicillin; n (%)	16 (19.3)	5 (9.6)
Amoxicillin-Clavulinic Acid; n (%)	23 (27.7)	20 (38.5)
Cefuroxime OR Cefprozil	14 (16.9)	11 (21.1)
Clarithromycin OR Azithromycin OR Erythromycin; n (%)	17 (20.5)	6 (11.5)
Both Amoxicillin AND (Clarithromycin OR Azithromycin); n (%)	4 (4.8)	3 (5.8)
Other; (%)	4 (4.8)	3 (5.8)

### Length of antimicrobial treatment

The median total length of antimicrobial therapy for all patients was 12 days (IQR 10 – 16). For those patients without effusion, the median length of treatment was 10 days (IQR 10 – 12), while those with effusion was 18.5 days (IQR 14 – 25).

## Discussion

This study is a review of pediatric CAP (diagnosed by consolidation on initial chest radiograph and compatible clinical history) at a pediatric hospital, two years after the introduction of universal PCV-7 pneumococcal vaccination. Previous reviews have had smaller sample size, were less comprehensive, were limited to developing nations, or were completed prior to the introduction of the conjugated seven valent pneumococcal vaccine [10,23-25].

Since the diagnosis of CAP is often based on a compatible clinical presentation and a CXR finding of consolidation, we chose these as practical requirements for study inclusion. Our sample selection was an attempt to increase the probability of capturing and evaluating bacterial pneumonias given that consolidation is most frequently associated with bacterial infection [11,26-28]. The small sample size of the radiographs that were reviewed by the radiologists likely resulted in the low kappa scores. These values are comparable to published results, and acknowledge the inter-observer variability in interpretation of chest radiographs in the absence of clinical information [11,28].

This study supports the significant continuing health care burden of children hospitalized with consolidative pneumonia in the era of PCV-7 use. [29] In this cohort, two-thirds of patients had at least one visit to a physician prior to their hospitalization and the average length of hospital stay was 4 days. One quarter of children had 3 or more CXRs, and nearly 20% had an invasive procedure done (chest tube insertion or VATS).

Our study found that patients presenting with effusion were not more likely to be younger, febrile, tachypneic or have a higher WBC count than those without effusion, underscoring the importance of chest radiography in pediatric CAP. Other authors have supported the importance of early radiologic evaluation in differentiating early complicated versus uncomplicated lobar pneumonia [25,30]. Children with an effusion were more likely to be admitted to the intensive care unit.

Only 9 of the 117 (7.7%) patients had bacterial pathogens identified in blood or pleural fluid. Seven had *Streptococcus pneumoniae* (6 in blood and 1 in pleural fluid) and 1 child had Group A *streptococcus* isolated from pleural fluid. The incidence of bacteremia was 6%, which is comparable to a recent study evaluating children with CAP in the Emergency Department [31], but lower than bacteremic rates published previously in the literature [12,32]. This is likely due to the fact that 42% of children in this cohort had antimicrobials prior to hospital presentation.

PCV-7 has been included in the publicly-funded routine immunization schedule in Ontario since 2005. By parental report, the majority of children had up-to-date vaccination series for age. Six of the seven *S. pneumoniae* serotypes identified in blood or pleural cultures were not PCV-7 serotypes, but would have been included in PCV-13 (Prevnar13®) or PCV-10 (Synflorix®) coverage. (4/6 children with PCV-13 vaccine serotypes would have been age-eligible for immunization and therefore had infection potentially preventable with PCV-13.) The only vaccine-preventable serotype (9 V) occurred in a 16-year-old youth who would not have received PCV-7 immunization. The only bacteremic serotype in this cohort not included in the 7, 10 or 13-valent vaccines was 11A.

Current pediatric CAP guidelines and reviews recommend consideration of treatment with intravenous ampicillin or penicillin for hospitalized children, given the evidence for its comparable effectiveness to cefuroxime for more severe pneumonia. [12-14,33-35]. Despite this, less than 5% of children received a penicillin as initial therapy. Only 19% of children with uncomplicated pneumonia received amoxicillin at discharge, and only 2 of the 7 patients with confirmed *S. pneumoniae* were discharged on amoxicillin. All *S. pneumoniae* isolates in this study were penicillin susceptible. (CHEO’s 2010 Antibiogram reports a 97% susceptibility of *S. pneumoniae* to penicillin [36].) Cephalosporins were prescribed in 96% of patients in this cohort rather than narrower-spectrum, less expensive antimicrobial agents, such as penicillin or ampicillin. We acknowledge that prior antimicrobial use may have influenced the physician’s antimicrobial choice on admission. Antimicrobial stewardship intiatives and recently published guidelines aimed at use of ampicillin or penicillin G (as opposed to broader spectrum coverage with second and third generation cephalosporins) may alter prescribing trends in the future.

Guidelines recommend empiric coverage with a macrolide in older children because *Mycoplasma pneumoniae* is more prevalent in this group [12-14]. More recent data has shown that *Mycoplasma pneumoniae* may play a more important role in the younger age group than previously believed [26]. Further studies may alter the current recommendations regarding the use of macrolides in pediatric CAP, however macrolide use should be cautioned in areas where there is significant pneumococcal resistance to macrolides. In this study, macrolides were used frequently in the younger age group - nearly half of children under age 5 years were treated with a macrolide either in hospital or at discharge.

The limitations of the retrospective nature and design of the study did not permit verification of specific immunization status for PCV-7. Microbiological data was also not available for all patients. Our inclusion criteria of consolidative pneumonia would have excluded patients with very early pneumonias that did not have established consolidation on initial CXR. The study results however, are generalizable to healthy children who are hospitalized for consolidative pneumonia in populations where PCV-7 is routinely administered to children.

## Conclusions

This study shows the substantial and continued morbidity associated with pneumonia in the era of universal PCV-7 vaccine coverage. Empiric antimicrobial choice is highly variable at this institution, emphasizing the need for clear evidence-based guidelines for the management of pediatric CAP.

## Competing interests

The authors declare that they have no competing interests.

## Authors’ contributions

AR-L participated in the study design and coordination, reviewed and extracted data from charts, and drafted the manuscript. NB participated in the design of the study and performed the statistical analysis. NLS conceived of the study, participated in its design and implementation, reviewed and extracted data from charts, and helped to draft the manuscript. NS and KK each independently reviewed a sample of the study CXRs. All authors read and approved the final manuscript.

## Pre-publication history

The pre-publication history for this paper can be accessed here:

http://www.biomedcentral.com/1471-2431/12/133/prepub
